# Potential of *Y. lipolytica* epoxide hydrolase for efficient production of enantiopure (*R*)-1,2-octanediol

**DOI:** 10.1186/s13568-023-01584-1

**Published:** 2023-07-26

**Authors:** Vijaya P. Godase, V. Ravi Kumar, Ameeta Ravi Kumar

**Affiliations:** 1 Biochemistry Research Laboratory, Department of Biotechnology (Formerly Institute of Bioinformatics and Biotechnology), Savitribai Phule Pune University, 411007 Pune, India; 2 Department of Biochemistry, Shivaji University, 416004 Kolhapur, India; 3 Chemical Engineering and Process Development Division, National Chemical Laboratory, 411008 Pune, India

**Keywords:** Deep Eutectic solvents, Optimization, Recombinant epoxide hydrolase, Response surface methodology, *Yarrowia lipolytica*

## Abstract

The recombinant Yleh from a tropical marine yeast *Yarrowia lipolytica* NCIM 3589 exhibited a high epoxide hydrolase activity of 9.34 ± 1.80 µmol min^-1^ mg^-1^ protein towards 1,2-epoxyoctane (EO), at pH 8.0 and 30 °C. The reaction product was identified as 1,2-Octanediol (OD) by GC-MS using EO and H_2_O^18^ as substrate, affirming the functionality of Yleh as an epoxide hydrolase. For EO, the *K*_m_, *V*_max_, and *k*_cat_/*K*_m_ values were 0.43 ± 0.017 mM, 0.042 ± 0.003 mM min^-1^, and 467.17 ± 39.43 mM^-1^ min^-1^, respectively. To optimize the reaction conditions for conversion of racemic EO by Yleh catalyst to enantiopure (*R*)-1,2-octanediol, initially, Response Surface Methodology was employed. Under optimized reaction conditions of 15 mM EO, 150 µg purified Yleh at 30 °C a maximal diol production of 7.11 mM was attained in a short span of 65 min with a yield of 47.4%. Green technology using deep eutectic solvents for the hydrophobic substrate (EO) were tested as co-solvents in Yleh catalyzed EO hydrolysis. Choline chloride-Glycerol, produced 9.08 mM OD with an increased OD yield of 60.5%. Thus, results showed that deep eutectic solvents could be a promising solvent for Yleh-catalyzed reactions making Yleh a potential biocatalyst for the biosynthesis of enantiopure synthons.

**Supplementary Information:**

The online version contains supplementary material available at 10.1186/s13568-023-01584-1.

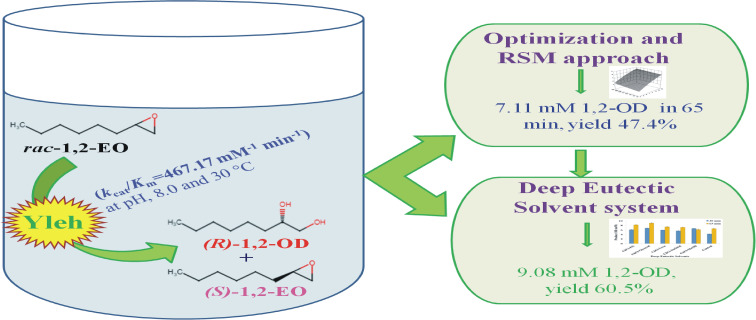

## Introduction

Vicinal diols and epoxides are important building blocks for the production of bioactive compounds used in cosmetics / personal care products and as raw materials for synthetic fiber, urethane compounds, water-based inks, insecticides, nanoparticles, etc. (Furuya et al. 2012; Effenberger et al. 2016; Sigg and Daniels 2020). Biological activity is highly stereoselective with the two enantiomers of the drug have different effects. Hence, regulatory authorities often warrant that chiral drugs be obtained optically pure to study pharmacological and toxicological properties. For example, EO and its vicinal diol, OD, are intermediates used in the asymmetric synthesis of high-value pharmaceutical compounds. The diol is an essential building block in the production of steroids, β-blockers, adrenaline, nerve protectants, HIV protease inhibitors, leukotriene, insect pheromones, in treating head louse infestations and for the production of ferroelectric liquid crystals (Archelas and Furstoss 1998, 2001; Otto et al. 1988; Genzel et al. 2002; Burgess et al. 2012).

Different methods have been used for the chemical synthesis of 1,2-octanediol using catalysts. Early processes for the preparation of vicinal diols and/or epoxides were carried out with unsaturated olefin compounds using alkali metals, alkaline earth metals, ammonium perrhenates, rhenium oxides as catalysts and hydrogen peroxide as an oxidation agent. Organic phosphoric acid esters and saturated ethers were used as solvents in pressure-controlled reactions occurring between 50 and 150 °C. The preparation of rhenium catalysts has its disadvantages with some of them being expensive and highly toxic. Additionally, yields of diols and epoxides were low with the formation of secondary oxidised products, such as ketones, carboxylic acids and/or polymeric condensation products (Warwel et al., 1994). Another common method to produce epoxy intermediates in the manufacture of 1,2-alkanediol can be carried out by combining 1-alkene with organic acid and hydrogen peroxide. Further, the epoxy compound is hydrolysed with alkali or an acid catalyst, followed by transesterification with alcohol to produce 1,2-alkanediol. However, the reaction is very slow and an alkyl organic acid ester may be generated as a by-product. Hence, higher concentrations of harsh chemicals, namely hydrogen peroxide, sulfuric acid, and benzene, are added to increase reactivity and lower by-product formation (Korean Patent KR102109133B1, 2020). A bifunctional Titanium silicalite (TS-1) catalyst system with both oxidative and Bronsted acid sites was designed by Prasetyoko (2006) for the consecutive transformation of 1-octene to octanediol with aqueous hydrogen peroxide as an oxidant. Another approach used active catalytic systems for alkene hydroxylation and cleavage by the highly toxic and expensive osmium tetroxide and transition metal catalysts such as ruthenium, iron, manganese, and cobalt to convert olefins into cis-1,2-diols. Palladium-catalyzed dihydroxylation and oxidative cleavage of olefins with oxygen as the sole oxidant and acid as an additive have also been studied (Wang and Jiang 2010). A more recent approach for obtaining diols from alkenes using electrolysis was studied using chemicals such as NaBr, THF, and Et_4_NBF_4_ with diol yields ranging from 10 to 56% but without enantioselectivity (Jud et al. 2021). Generally, these 1,2-diols are prepared by chemical processes having several limitations, as mentioned above, namely, the use of toxic chemicals, low substrate-to-catalyst ratios, and low efficiency with reactions carried out under extremes of temperature and pressure and waste generation. In this connection, mild, green, and clean processes such as the biocatalytic hydrolysis of racemic epoxides by epoxide hydrolases (EC 3.3.2.3) may prove advantageous.

Amongst eukaryotic microbes, EHs from a few fungal/yeast sources have been studied in detail, e.g., *Aspergillus niger* (Arand et al. 1999), *Trichoderma reseei* (Oliveira et al. 2016), *Rhodotorula glutinis*, *Rhodosporidium toruloides* and *Saccharomyces cerevisiae* (Smit 2004; Elfstrom and Widersten 2005). They exhibit different substrate specificities and enantio preferences, which are enzyme-dependent. Most of these microbial enzymes show very low selectivity towards 1,2-epoxyoctane except for *R. araucariae* CBS 6031 and *R. toruloides* CBS 0349, with 43 and 47% yield for diol, respectively (Botes et al. 1998).

One of the major challenges in enantioselective catalysis using these enzymes is the low water solubility of substrates, low product formation, side product formation due to limited mass transfer, and incomplete conversion due to kinetic or thermodynamic inhibition. Generally most enzymes have lower activity and stability in organic / non-aqueous systems which frequently serve as a limitation. Factors such as diffusion, low substrate solubility, stabilization of enzyme-transition state complex, removal of bound water and change in enzyme conformation are responsible for lower stability and activity of enzymes (Wang et al. 2016). For example, subtilisin had reduced catalytic efficiency but higher stability in octane as compared to water. Similarly α-chymotrypsin had reduced activity of 27 and 33% in 1-and 2-propanol, respectively as compared to control. Molecular Dynamics simulation studies have shown that lysozyme has reduced enzyme activity in different concentrations of methanol and hexane (Mohtashami et al. 2019). Hence, to extend the lifetime of enzymes in the presence of organic/ non-aqueous solvents, strategies such as engineering of enzymes are being applied (Stepankova et al. 2013; Steenkamp 2021). One can also increase the solubility of water-immiscible reactants and suppress the undesired hydrolytic reactions by using aqueous solutions containing water-miscible organic or non-aqueous solvents. Such green and sustainable alternatives to hazardous organic solvents are Ionic liquids (ILs) and Deep Eutectic Solvents (DES). They have several useful properties: non-flammability, high solvation ability, and negligible vapour pressure. However, ILs, with their high costs, complex synthesis method, and scarce information on their toxicity, are not considered ideal for large-scale use. DESs are mixtures of constituents that are primary metabolites like amino acids, alcohols, sugars, or choline derivatives (Paiva et al. 2014). They are inexpensive to prepare, can be easily purified, are biocompatible with enzymes, and are biodegradable and more environmentally friendly than ILs (Schweiger et al. 2019). They can easily be obtained by mixing different organic compounds, which are hydrogen acceptors and hydrogen-bond donors, thereby influencing their solvation properties. For example, the hydrolysis of (1,2)-trans-2-methyl-styrene oxide catalyzed by potato epoxide hydrolase in DES significantly increased substrate solubility and product yield (Lindberg et al. 2010).

In the present study, we have optimized the reaction condition parameters for the increased production of the hydrophobic substrate *rac*1,2-epoxyoctane to (*R*)-1,2-octanediol by the highly active recombinant epoxide hydrolase (Yleh) from the yeast *Y. lipolytica* NCIM 3589, enabling its usage in sectors like industry and enzyme engineering.

## Materials and methods

### Chemicals, strains, media and growth conditions

Media components and supplements were purchased from HiMedia, Mumbai (India). Solvents and compounds were procured from Merck, Mumbai (India), Sisco Research Laboratory, Mumbai (India), and Sigma Aldrich (USA). Racemic 1,2-epoxyoctane, racemic 1,2-octanediol, (*R*)-1,2-epoxyoctane, (*R*)-1,2-octanediol, Choline chloride were procured from Sigma Aldrich (India), Trifluoroacetic anhydride (TFA) from HiMedia (Mumbai, India), Dichloromethane (DCM) and Ethyl acetate (EtAc, HPLC grade), Ethylene Glycol (EG), Glycerol was obtained from Merck (HiMedia, India), anhydrous sodium sulfate Sorbitol, Urea, was from SRL (SRL, India). Nitrogen (UHP Grade, 99.999%), Hydrogen (99.999%), and Zero Air (dry air, > 99.995%) gas cylinders were supplied by a local supplier. 1 mL screw-cap vials with rubber inserts provided by Agilent Technologies (India), 3 mL screw-cap vials with rubber inserts supplied by a local distributor. Standard stock and working solutions (the concentration range from 1 to 100 mM) of EO and OD for standard curves were prepared in ethyl acetate-HPLC grade and stored at 4 °C and room temperature, respectively. All the compounds were > 99% pure as per the manufacturer’s specifications.

For cloning and expression, *Escherichia coli* DH5α and *E. coli* BL21AI (Invitrogen, USA) were used, respectively. Plasmid vector pET28a+ (Novagen, USA) used for molecular work. The *E. coli* strains were routinely cultured and maintained on Luria-Bertani (LB) medium broth or agar supplemented with Kanamycin (50 µg/mL) when necessary at 37 °C for 16 h at 120 rpm.

### Cloning, expression and purification

The recombinant Yleh from *Y. lipolytica* was cloned, expressed, and purified as given in Supplementary Files. The Bradford method estimated the protein concentration, and SDS-PAGE evaluated the purity and molecular weight of the protein.

### Enzyme activity assay and product identification

EH activity was analyzed spectrophotometrically using EO as substrate by measuring residual epoxide at 590 nm with 4-(p-nitrobenzyl) pyridine (pNBP), according to the previously reported protocol (Bendigiri et al. 2017). One unit (U) of enzyme activity is defined as the amount of enzyme catalyzing the conversion of 1 µmol of EO per min under the assay conditions. All the assays were carried out thrice in triplicates unless otherwise stated.

For product identification, conversion of EO to its corresponding product 1,2-OD was carried out by reacting 5 mM EO with 50 µg of purified Yleh in 50 mM Tris-sulfate buffer, pH 8.0, and incubated at 30 °C for 1 h under nitrogen-purged conditions to eliminate aerial and dissolved oxygen. The product was extracted with ethyl acetate, concentrated by drying, and injected on a DB624 column (30 m×0.25 mm×0.25 μm) connected to GC-MS (Agilent Technologies, USA) using nitrogen as carrier gas. The program used for analysis was as follows: initial temperature 60 °C held for 30 s, 15 °C/min up to 120 °C held for 30 s and 30 °C/min up to 240 °C, and held for 5 min. The product was identified by GC-MS using the NIST Mass Spectral Library (NIST05).

### Labeled water (H_2_O^18^) experiment

An experiment using labeled water (H_2_O^18^) was conducted to confirm the incorporation of oxygen into the reaction product diol from the water molecule. For this, 50 µg Yleh protein was incubated with 5 mM 1,2-EO at 30 °C for 1 h in 1 ml 50 mM Tris-sulphate buffer, pH 8.0, and an equal volume of labeled water (H_2_O^18^). Trace-Ultra GC (Thermo Fisher Scientific, USA) was used for GC-MS analysis. The sample was then centrifuged and extracted with ethyl acetate (1:1 v/v). It was further concentrated by drying and injected on a DB-5 column (30 m x 0.25 mm x 0.25 mm, Agilent Technologies, USA) connected to GC-MS and a mass detector (ITQ 1100, Thermo Fisher Scientific, USA) with helium as a carrier gas. The program used for analysis was as follows: initial temperature 60 °C, 15 °C/min up to 160 °C held for 30 s and 3 °C/min up to 200 °C and held for 1 min. The reaction product peaks were identified by *m/z* values and retention time using NIST Mass Spectral Library (NIST 05).

### Effect of pH, temperature and additives on Yleh activity

The effect of pH, temperature, and additives on the epoxide hydrolase activity of recombinant Yleh was determined as mentioned in the Supplementary information.

### Substrate specificity studies for epoxide hydrolase activity

The substrate specificity of Yleh was studied by measuring the epoxide hydrolase activity toward various terminal epoxides (aliphatic epoxy compounds). All reactions were conducted in 50 mM Tris-sulphate buffer, pH 8.0 at 30 °C with 1 mM compounds and expressed as relative activity (%) considering activity with EO as 100%.

### Kinetic properties of Yleh with EO

The effect of EO concentration (0.1–0.7 mM) on EH activity was assessed using purified Yleh (1 µg) in 50 mM Tris-sulphate buffer, pH 8.0. Yleh activity was determined at optimum conditions as described above. The kinetic constants values, *K*_m_ and *V*_max_, were calculated using the Michaelis-Menten equation in the Lineweaver-Burk plot. The turnover number or catalytic constant (*k*_cat_) was determined by the equation *k*_cat_ = [*V*_max_/E_T_], where *V*_max_ is the maximal velocity, and E_T_ is the total enzyme concentration used in the assay. The catalytic efficiency or specificity constant (*k*_cat_/*K*_m_) was also determined. Using OriginPro 8 SRO software (OriginLab Corporation, USA), the data analysis was carried out by non-linear regression.

### Biocatalytic hydrolysis of 1,2-EO with recombinant Yleh in an aqueous system

An aqueous buffer system with 2 mL of Tris-sulphate buffer, pH 8.0 containing EO substrate (5 mM), and purified Yleh enzyme (50 µg) was allowed to react in glass test tubes covered with caps at the selected temperatures. At a given time, stopped the reaction and products extracted with an equal volume of ethyl acetate (1:1 v/v), concentrated by drying, and subsequently analyzed by GC as mentioned in the experimental section.

### GC analysis for detection of extracted product 1,2-octanediol

The enzyme assay was performed with racemic EO and the recombinant Yleh in 50 mM Tris-sulphate buffer, pH 8.0. The reaction products were extracted with ethyl acetate (1:1 v/v). GC analysis was carried out using a non-chiral RTx-5 capillary column (30 m x 0.25 mm x 0.25 μm, Restek Corporation, USA) using Shimadzu GC-2014 system (Shimadzu, Japan) equipped with a flame ionization detector (FID). The column temperature was set at 40 °C for 2.0 min, increased linearly to 240 °C at 25 °C/min, and held for 4 min. The temperature of the injector and detector were set at 180 and 250 °C, respectively, and nitrogen was used as a carrier gas (linear velocity, 16 cm/s). For the standard curve, the stock solution of OD (50 mM in ethyl acetate) was prepared and used the working concentration range (1–50 mM) to analyze the area under the curve by non-chiral phase gas chromatography, as given below. A plot of standard OD concentration in mM versus the average area under the curve was plotted, and the linear fit obtained was used for analysis. The retention time of standard racemic OD and EO were 11.104 and 4.912 min, respectively.

Standard compounds and the extracted reaction product were derivatized and analyzed for chiral phase GC analysis on the chiral column. Briefly, 17 mM standard *rac*-OD and (*R*)-OD were added to 2 mL of DCM in individual screw-cap vials. 200 µL of trifluoroacetic anhydride (TFA) was added and heated at 100 °C for 20 min. After this, removed caps and vials were carefully evaporated using dry nitrogen gas. The residual part from the vials was dissolved in 1 mL DCM, and 1 µL sample was injected into the chiral column. The enzyme assay was performed in 50 mM Tris-sulphate buffer, pH 8.0, using 50 mM each *rac*-EO and (*R*)-1,2-EO as substrates with 75 µg purified Yleh protein and incubated at 30 °C for 65 min. The reaction product was extracted, as mentioned earlier. Further, the extracted product was dried using nitrogen gas and derivatized, as discussed above. The analysis was carried out by chiral phase GC with Shimadzu GC-2014 system (Shimadzu, Japan) equipped with a flame ionization detector (FID) and GC-MS/MS on Shimadzu TQ 8030 (Shimadzu, Japan). The chiral GC column, Astec®-Chiraldex G-TA fused silica C capillary column (30 m x 0.25 mm x 0.12 μm, Supelco Analytical, Sigma, USA) was used. The column temperature was set at 80 °C for 2.0 min, increased linearly to 130 °C at 5 °C/min, and held for 8 min. The injector and detector temperatures were set at 250 °C, and nitrogen was used as the carrier gas (linear velocity, 44.3 cm/s).

### Statistical design of experiments

#### Experimental design software

To collect the response data for the production of OD, two-level factorial and three-level factorial design matrices were generated and studied by Design-Expert® software version 7.1.3 (Stat-Ease Inc. Minneapolis, USA).

#### Two-level factorial design of experiments

The two-level factorial technique is used to ascertain the subset of factors that control the process response by investigating a set of n variables (Myers et al. 1995, 1997; Montgomery and Runger 2009). Since the number of factors (variables) was few for this study, we used a traditional two-level factorial experimental design. It provides an effective means to screen the factors and select the critical ones. It also permits the estimation of all significant effects and dominant interactions.

The factors chosen for the study included substrate concentration, enzyme concentration, reaction temperature, and reaction time are essential variables in the enzyme reaction resulting in OD production. The study involved 8 different experimental runs for 4 chosen factors likely to be significant at low (-1) and high (+ 1) coded levels, as given in Table 1. The reaction mixture (2 mL) in 50 mM Tris-sulphate buffer, pH 8.0, was incubated at the chosen temperature (°C) and reaction time (min) levels, as mentioned in Table 1. After the termination of the reaction, OD was extracted with equal volumes of ethyl acetate (1:1 v/v), and the organic layer was separated and dried with anhydrous sodium sulfate. The extracted reaction products were reconstituted in 500 µL ethyl acetate and estimated by GC. The experiments were performed in duplicate and used the mean response for analysis using the Design of Experiments to identify the factors and make inferences. The study was carried out by placing a reduced two-level factorial interaction model. Analysis of Variance (ANOVA) was performed using the proposed model, and the R2 coefficient of determination determined the best-fit model. The statistical significance of the model was tested by F-test, and based on the standardized effects plot, arranged the factors into two sets, namely, those that can be considered optimized for OD production and those that could be further optimized. To determine the significant variable(s) based on confidence levels above 95% (p < 0.05) studied, ANOVA analysis of the selected factorial model.

**Table 1:** Experimental factors and chosen levels of variables used for two-level factorial design

Factors	Coded	Levels	
		-1	+ 1
Substrate (mM)	A	2.5	25
Enzyme (µg)	B	2	100
Temperature (°C)	C	25	45
Reaction time (min)	D	10	120

#### Three-level factorial design of experiments

The three-level factorial design studies the interaction between the variable and response using a quadratic model and requires factors to be run at three chosen levels (-1, 0, + 1) (Myers et al. 1995, 1997; Montgomery and Runger 2009). This experimental design has fewer runs as compared to the other three-level factorial methods. It is more favorable and less expensive to perform compared to Central Composite Design (CCD) for the same number of variables. The factors chosen for optimization by three-level factorial design after two-level factorial design are given in Table 2, and it involved 13 runs for the two selected factors as variables. The OD response obtained was analyzed by Design-Expert® software to fit a polynomial regression equation best as follows:

**Table 2:** Experimental levels, values of variables and constant factors used in three-level factorial design

Variable Factors	Coded	Levels		
		-1	0	+ 1
Substrate (mM)	A	1	8	15
Enzyme (µg)	B	75	112.5	150
**Constant Factors**			**Value**	
Temperature (°C)	C		30	
Reaction time (min)	D		65

1 $$\begin{array}{*{20}{c}}{y = {\beta _0} + \sum {{\beta _i}{X_i}} + \sum {{\beta _{ij}}{X_i}{X_j}} + \sum {{\beta _{ii}}{X_i}^2i} }\\{ = 1,2,j = 1,2,i \ne j}\end{array}$$

Where *y* is the response measured, *β*_*0*_ is the intercept, *X*_*i*_ and *X*_*j*_ are the independent factors, *β*_*i*_ are the linear coefficients, *β*_*ii*_ are the quadratic coefficients, and *β*_*ij*_ are the interaction coefficients.

ANOVA was studied for the response of the factorial model to determine the significance of the variables based on confidence levels above 95% (p < 0.05). From the R^2^ value, the model fit was ascertained, while the F-test was used to determine the statistical significance. Three-dimensional response surface plots were studied to visualize the response interactions for the levels of the variables.

#### Time course studies for OD production

A time course study was carried out to validate response surface methodology data to check the process behavior under optimized conditions. A separate reaction was carried out for each time point, and the product was extracted, as mentioned in the experimental section.

### Effect of deep eutectic solvents on epoxide hydrolase activity and OD production

#### Preparation of deep eutectic solvents

Deep Eutectic Solvents choline chloride/ethylene glycol (ChCl-EG, 1:2 mol/mol), choline chloride/glycerol (ChCl-Glycerol, 1:2 mol/mol), choline chloride/urea (ChCl-urea, 1:2 mol/mol), choline chloride/sorbitol (ChCl-Sorbitol, 1:1 mol/mol), choline chloride/methanol (ChCl-MeOH, 1:2 mol/mol) was prepared by gently heating and stirring the corresponding individual components for 60 min at 60–80 °C until a clear solution was obtained.

#### Hydrolysis of EO with Yleh in Deep Eutectic solvents containing system

In the Deep Eutectic Solvents containing system, hydrolysis of EO to its corresponding product was carried out with optimized concentrations of substrate and enzyme in capped glass test tubes by reacting EO (15 mM) with purified Yleh (150 µg) in 1 mL of 50 mM Tris-sulfate buffer, pH 8.0 containing 15% (v/v) of the above mentioned Deep Eutectic Solvents. The reactions were incubated at 30 °C for two-time points, i.e., 30 and 65 min, with their respective controls and the products extracted and analyzed as mentioned earlier. The hydrolysis yields were calculated according to the following equation,

2 $${Y_{OD}}\left( \% \right){\rm{ }} = {\rm{ }}\left( {{C_{OD}}/{C_{initial{\rm{ }}EO}}} \right){\rm{ }}x{\rm{ }}100.$$

For each experiment, controls were included in which the abiotic hydrolysis was subtracted from the total hydrolysis to obtain the enzymatic hydrolysis.

#### Absolute configuration of epoxide 1,2-epoxyoctane and diol 1,2-octanediol

The absolute configurations were determined for the residual EO and the product OD obtained by this hydrolytic enzyme reaction. Briefly, 100 mM 1,2-EO (100 mg) and 100 µg recombinant Yleh enzyme were incubated in 10 mL 50 mM Tris-sulphate buffer, pH 8.0, and the reaction mixture was incubated at 30 °C for 1 h with gentle shaking. The reaction was stopped, and the remaining 1,2-EO was extracted with an equal volume of chilled pentane (Weijers et al. 1998). The organic layer was dried with MgSO_4_ salt and concentrated by evaporation to measure the specific optical rotation values.

For the absolute configuration of the reaction product (1,2-OD), from the same hydrolytic reactions, the remaining aqueous reaction mixture was extracted twice with an equal volume of ethyl acetate (1:1 v/v). The organic layer was dried with MgSO_4_ salt and evaporated under reduced pressure to give an oily residue of product OD. The concentrated diol was re-dissolved in methanol and used to measure specific optical rotation values. Optical rotation values were measured on a P-2000 Jasco Polarimeter at 589 nm, and data were analyzed using the Spectra Manager™ software provided with the instrument.

### Statistical analysis

All experiments were performed in duplicate, and the data were expressed as mean ± SEM. As mentioned in earlier sections, analysis for the two-level factorial and 3-level factorial design experiments was carried out.

## Results

### Cloning, expression and purification of Yleh

Yleh, *Y. lipolytica* epoxide hydrolase with an ORF of 1065 bp (Gene Bank Accession number XP_499652.1) encodes a polypeptide of 354 amino acid residues; was overexpressed in *E. coli* BL21AI cells and purified to > 95% pure homogeneity using Ni-NTA affinity chromatography followed by size exclusion chromatography with a yield of 19 ± 0.5 mg/L. This recombinant protein showed a single band on SDS-PAGE (Fig. S1).

### Enzyme activity and product identification

The recombinant protein Yleh exhibited a high EH activity of 9.34 ± 1.80 µmol min^− 1^ mg^− 1^ protein with EO as substrate and seemed to have one of the highest EH activities amongst fungi reported to date.

**Fig. 1:**
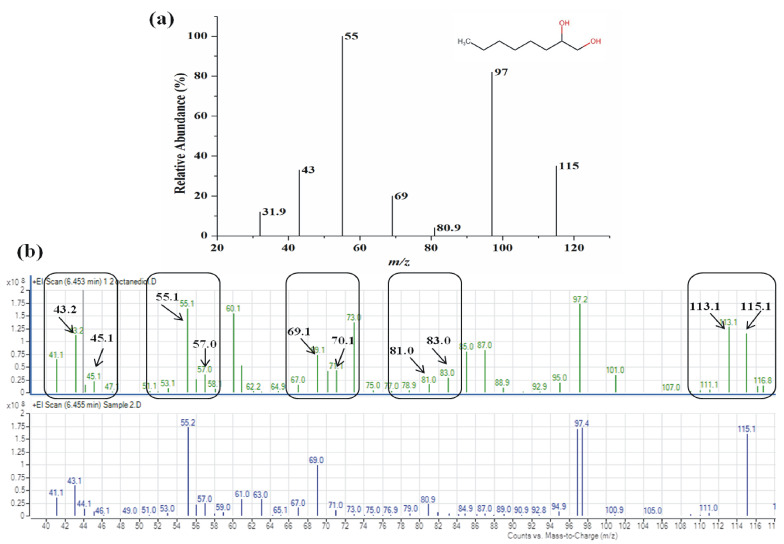
Mass spectroscopy analysis for enzymatic reaction product identification

The enzymatic reaction product identification was carried out by GC-MS analysis using EO as substrate. The product eluted at RT = 8.980 min with *m/z* = 146 and exhibited daughter fragments of *m/z* (M-H+) 31.9, 43, 55, 69, 80.9, 97, 115 (Fig. 1a). The best hits obtained were for 1,2-octanediol with NIST Mass Spectral Library (NIST 05) confirming EH activity of recombinant Yleh.

### Labeled water (H_2_O^18^) experiment

As mentioned in the experimental section, an enzyme assay was carried out using O^18^-labeled water. In studies with labeled water (H_2_O^18^), a single peak eluted at RT = 6.455 min, and no other peak could be identified under the experimental conditions. The GC-MS spectra shown in Fig. 1b stated the ion fragmentation pattern of the peak, which showed the presence of fragments at *m/z* = 43.2, 45.1, 55.1, 57.0, 69.1, 71.1, 85, 87, 97.2, 115, 116.8 (Fig. 1b) suggesting the incorporation of O^18^ from H_2_O^18^ into 1,2-octanediol. Thus, GC-MS analysis suggests that the incorporation of O^18^ from the labeled water occurs into 1,2-octanediol by a hydrolytic mechanism of Yleh.

### Effect of pH, temperature and additives on Yleh activity

With EO as the substrate, the enzyme was found to be active in the pH range of 6–10 and exhibited maximal activity at pH 8.0 with the optimal temperature at 30 °C (Fig. S2).

To determine the effect of additives on Yleh activity, different metal salts, chemicals, reducing agents, and coenzymes (1 mM) were used (Table S1), and an enzyme assay was carried out with EO substrate. The reducing agents DTT and 2-mercaptoethanol, NADH and NADPH coenzymes, and metal chelator EDTA showed no significant effects on the EH activity of Yleh. The metal ions, Ag^2+^, Hg^2+^, and Zn^2+^ salts strongly inhibited the Yleh activity, Fe^2+^, Ca^2+^, and Ni^2+^ partially inhibited, and Fe^3+^, Mn^2+^ showed no effect on the enzyme activity. The enzyme activity increased in the presence of metal ions Cu^3+^ and Cu^2+^.

### Substrate specificity studies for epoxide hydrolase activity

To investigate the substrate specificity of the Yleh towards terminal epoxides, different epoxy compounds were screened for EH activity. Figure S3 shows the relative enzyme activity (%), demonstrating the broad substrate specificity of the Yleh towards terminal epoxides. The enzyme hydrolyzed terminal epoxides with aliphatic chains varying in carbon chain length (C_4_-C_12_), i.e., 1,2-epoxybutane (EB) to 1,2-epoxydodecane (EDD) to varying degrees and no catalytic activity towards these substrates was detected in the acidic pH range. Yleh hydrolyzed monosubstituted epoxides at the C1 position with an aliphatic chain with maximal activity towards 1,2-EO at pH 8.0. The EH activity increased as the chain length increased from 1,2-epoxybutane to 1,2-epoxyhexane. In contrast, no activity was seen for epichlorohydrin (ECH) and cyclic styrene oxide under the given assay conditions.

### Kinetic properties of Yleh with EO

The kinetic parameters, *K*_m_, and *k*_cat_ of the recombinant Yleh were determined with substrate EO using Michaelis-Menton and Lineweaver-Burk equations (Fig. S4). The recombinant Yleh showed a *K*_m_ value of 0.43 ± 0.017 mM with *V*_max_ of 0.042 ± 0.003 mM min^− 1^as seen by the L-B plot. The *k*_cat_ catalytic constant was found to be 200.75 ± 12.98 min^− 1^ for EO. The specificity constant, i.e., catalytic efficiency (*k*_cat_/*K*_m_) of Yleh for EO determined was 467.17 ± 39.43 mM^− 1^ min^− 1^. A Hill coefficient of 1.152 ± 0.064 was calculated, suggesting a single EO is associated per catalytic subunit.

### Statistical design of experiments

Since the enzyme exhibited a good turnover rate towards EO, leading to the industrially relevant OD, experiments were conducted to optimize the conditions for its production. The linear fits obtained for the standard curve of OD ascertained the concentrations of OD in the experimental samples.

### Optimization by the two-level factorial design experiments

Four factors at their low (-1) and high (+ 1) levels were selected as process parameters for optimizing OD production, as mentioned in Table 1. The results for diol production for the 8 runs are given in Table 3. The highest and lowest OD amounts obtained were in run 5 (2.474 mM OD) and run 3 (0.017 mM OD), respectively. The response values obtained were analyzed after the complete set of two-level factorial runs.

**Table 3:** The OD yield response values obtained for the Yleh catalyzed reaction from two-level factorial experiments

Run	A	B	C	D	Response
	Substrate	Enzyme	Temperature	Reaction Time	OD
	(mM)	(µg)	(°C)	(min)	(mM)
1	25	2	25	120	0.026
2	25	100	45	120	2.258
3	2.5	2	25	10	0.017
4	25	2	45	10	0.047
5	25	100	25	10	2.474
6	2.5	100	25	120	0.218
7	2.5	2	45	120	0.024
8	2.5	100	45	10	0.454

The best-fit two-factor interaction model in terms of actual factors was obtained as follows:

3 $$\begin{array}{*{20}{c}}{OD = + 0.01685 - - 1.11565E - 03*substrate}\\{ + 9.35941E - 04*enzymeconcentration}\\{ + 9.13379*substrate*enzymeconcentration}\end{array}$$

The above equation, in terms of actual factors, can make predictions of the response in original units for a given level of each factor.

The best-fit model in terms of coded factors can also be obtained as follows:

4 $$OD{\rm{ }} = {\rm{ }} + 0.69{\rm{ }} + {\rm{ }}0.51*A + {\rm{ }}0.66*B{\rm{ }} + {\rm{ }}0.50*AB$$

The above-coded equation may identify the factors’ relative impact by comparing the factor coefficients. The results were analyzed for ANOVA, and the best-fit model F value of 197.56 implies that the model is significant. There is only a 0.01% chance that an F value this large could occur due to noise. Values of “Prob > F” < 0.05 indicate the model terms to be significant. A (substrate), B (enzyme concentration), and AB are substantial model terms here. “Prob > F”> 0.1 indicate that the model terms are not significant. The “Pred R^2^” of 0.9732 is in good agreement with “Adj R^2^” of 0.9883. “Adeq Precision” measures the signal-to-noise ratio, and a ratio > 4 is recommended. The obtained ratio of 29.256 shows the presence of an adequate signal. The model, therefore, can be used to navigate the design space.

**Fig. 2:**
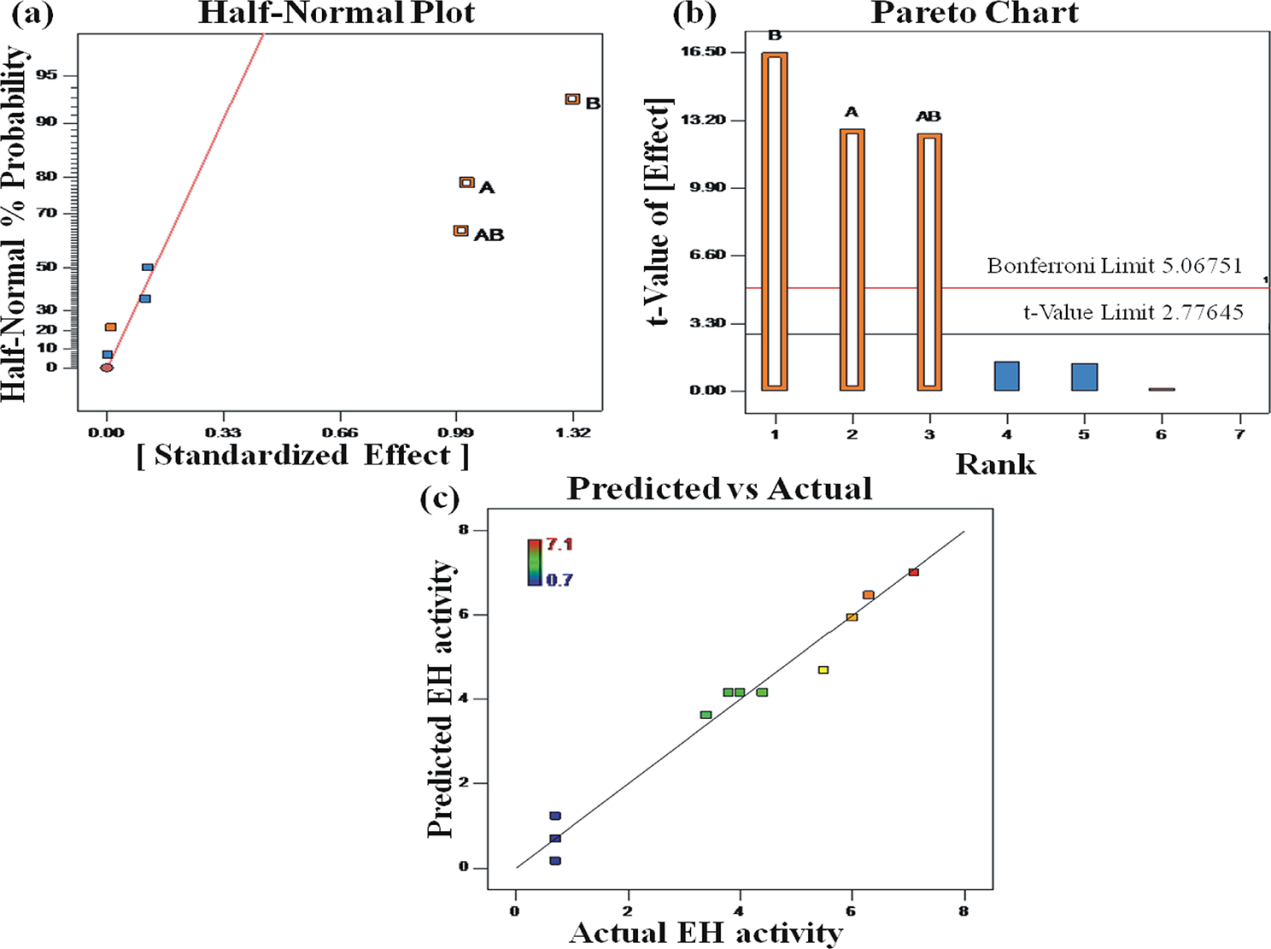
Studying the effects of factors on OD production from two-level factorial experiments. **(a)** Half-normal plot. **(b)** Pareto plot. Factors showing positive effects are indicated in orange colour, and those showing negative effects are indicated in blue colour. **(c)** Diagonal plot of predicted vs. actual values for the OD production using a three-level factorial design

The resulting Half-Normal probability plot is shown in Fig. 2. It is a graphical tool that puts into order estimated effects to help assess which factors are important and those which are unimportant. The results clearly show that the variables with positive outcomes are substrate concentration (A), enzyme concentration (B), and a two-factor interaction term (AB) of the product of enzyme and substrate concentration. The remaining terms, reaction temperature (°C) and reaction time (min) are less significant and form a linear relationship.

The results were also analyzed by studying the Pareto plot (Fig. 2b) to confirm that no more significant effects were apparent in the half-normal plot. Selected effects above the Bonferroni limit (value of 5.06752) are essential and should be included in the model. Effects above the t-value limit (value of 2.77645) are significant, and the experimenter should make a decision. Effects that are below the t-value need not be selected. In the present study, the Pareto plot corroborates the ANOVA and half-normal plot results. Therefore the factors chosen for further optimization were A-substrate and B-enzyme concentration.

### Optimization by the three-level factorial design experiments

The three-level factorial design with 13 runs was generated using the Design-Expert® software for the two factors identified (substrate and enzyme concentration) from two-level factorial design studies (Table 2). The reaction temperature and time factors were kept at their mean values (30 °C and 65 min) of the chosen range. The three-level factorial designs have three levels for each factor and are fit to a quadratic model. After conducting the experiments, the results obtained for OD production are presented in Table 4. The highest diol production was obtained in run 13 (7.1 mM OD), while the lowest diol production was obtained in runs 2 and 3 (0.7 mM OD).

**Table 4:** Experimental design and the OD production obtained by three-level factorial design

Run	A	B	Response
	Substrate (mM)	Enzyme (µg)	OD (mM)
1	8	112.5	4.0
2	1	75	0.7
3	1	150	0.7
4	15	112.5	6.3
5	8	112.5	4.4
6	15	75	6.0
7	1	112.5	0.7
8	8	112.5	3.8
9	8	75	3.4
10	8	112.5	4.0
11	8	150	5.5
12	8	112.5	4.0
13	15	150	7.1

The results obtained were analyzed for ANOVA in a fashion similar to the studies using the three-level factorial design, as shown in Fig. 2c.

The best-fit three-level quadratic model in terms of actual factors was obtained as follows:

5 $$\begin{array}{*{20}{c}}{OD = - 1.48756 + 0.59927*substrate + }\\{0.01422*enzyme - - 0.011710*substrat{e^2}}\end{array}$$

while the best-fit model in terms of coded factors was also obtained as follows:

6 $$OD{\rm{ }} = {\rm{ }} + 4.14{\rm{ }} + {\rm{ }}2.88*A + {\rm{ }}0.55*B{\rm{ }} - {\rm{ }}0.57*{A^2}$$

The above-coded equation may again be used to identify the factors’ relative impact by comparing the factor coefficients. The best-fit model F value of 100.15 implies that the model is significant. There is only a 0.01% chance that an F value this large could occur due to noise. Values of “Prob > F” < 0.05 indicate the model terms to be significant. In this case, A (substrate), B (enzyme concentration), and A^2^ (substrate^2^) are significant model terms. “Prob > F”> 0.1 indicate that the model terms are insignificant. The “Pred R^2^” of 0.922 is in good agreement with R^2^ = 0.9707 and “Adj R^2^” of 0.9612. The obtained “Adeq Precision” value of 29.428 shows the presence of an adequate signal. The quadratic model, therefore, can be used to navigate the design space.

### Response surface analysis

**Fig. 3:**
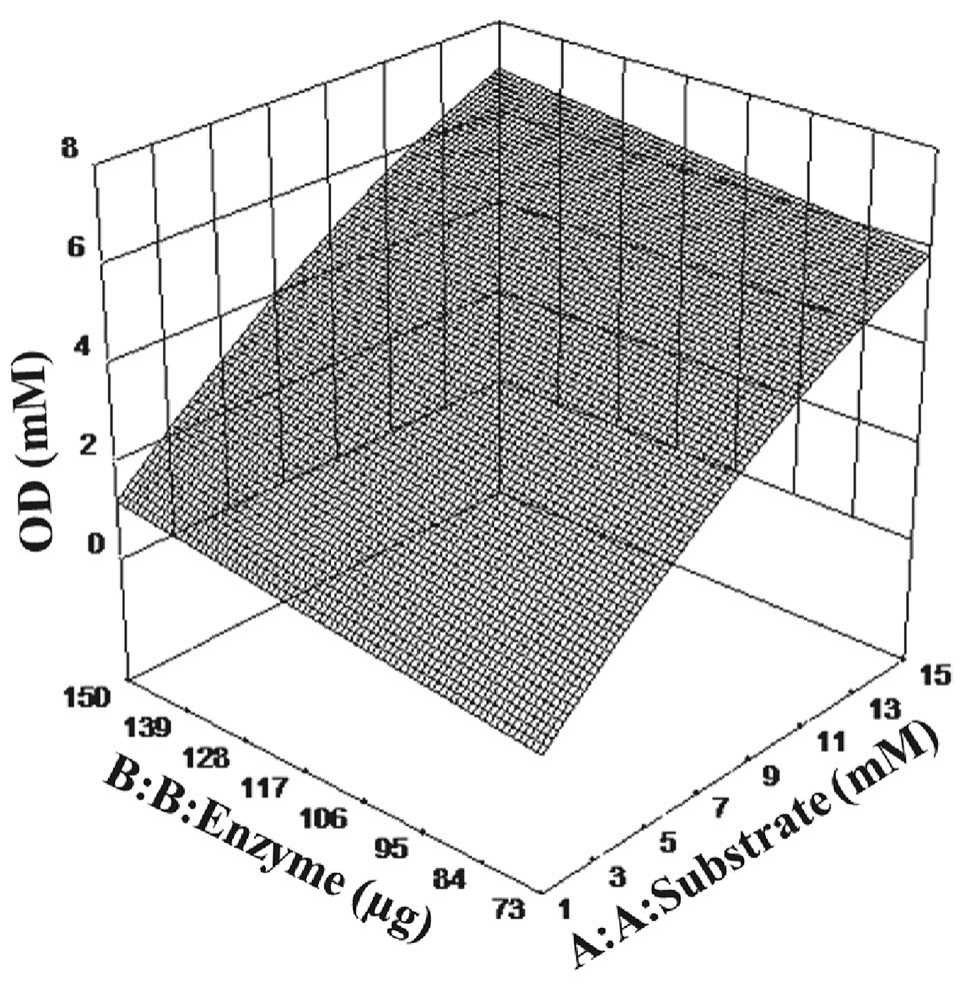
3-D response surface showing the overall two-factor interactions between enzyme and substrate concentration on OD production

Three-dimensional response surface plots were drawn to study the nature of the interactions between substrate and enzyme concentration and their effects on OD production. Figure 3 shows the non-linear nature of the interactions arising from the quadratic term A^2^ in the model. Therefore, further optimization studies in three-level factorial experiments have complemented the results of two-level factorial studies. The discussed response surface methodology involving two optimization steps has led to ascertaining the design space for enhanced OD levels.

### Time course study for OD production

**Fig. 4:**
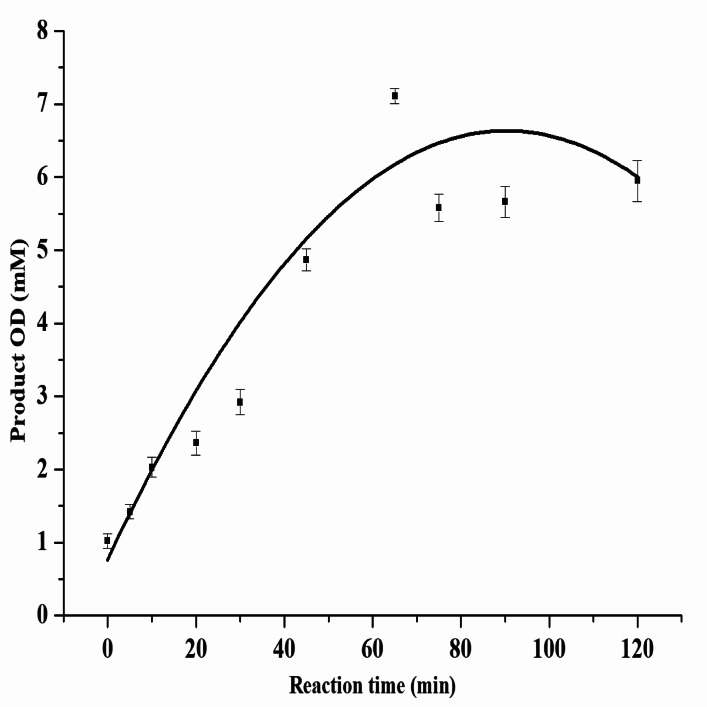
Time course for 1,2-octanediol production in optimized reaction conditions. All the experiments were performed with 3 independent sets with each set in triplicate and data were expressed as mean (± SEM.)

For the production of OD in the optimized concentrations of substrate and enzyme, the time course was monitored for 120 min. As shown in Fig. 4, maximal diol production of 7.11 mM was achieved in 65 min with a yield of 47.4%. An unoptimized enzyme assay produced only 0.02 mM OD. Thus, independent validation experiments under optimized reaction conditions showed reproducible results. Substrate EO, 15 mM; purified Yleh enzyme, 150 µg; reaction incubation conditions at 30 °C for 65 min found the optimized reaction conditions.

### GC analysis for detection of extracted product 1,2-octanediol

GC was efficiently used to detect the reaction products after enzymatic hydrolysis of racemic EO substrate. From GC-MS analysis, the product was identified as 1,2-octanediol and then followed up by chiral GC analysis. The chiral-GC analysis showed that the reaction product was (*R*)-1,2-octanediol with a retention time of 13.562 min in comparison with a standard (*R*)-1,2-octanediol, 13.558 min.

### Effect of Deep Eutectic solvents on epoxide hydrolase activity of Yleh and 1,2-Octanediol production

**Fig. 5:**
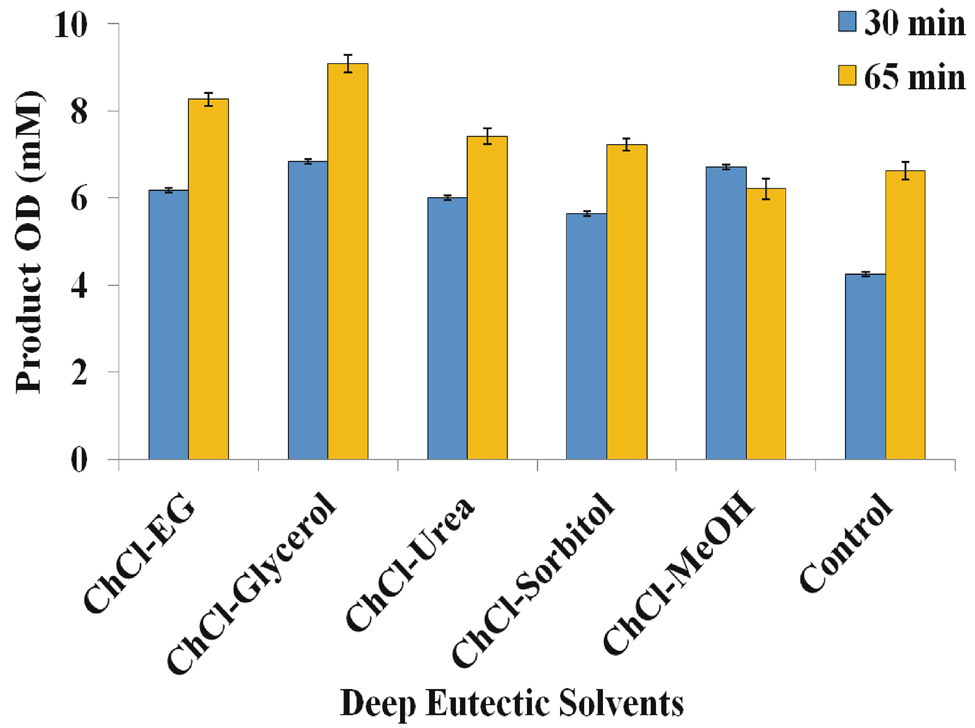
Effect of Deep Eutectic Solvents on OD production. All the experiments were performed with 3 independent sets with each set in triplicate and data were expressed as mean (± SEM).

Due to the limited solubility of EO in aqueous media, we studied the suitability of conducting enzymatic hydrolysis in eutectic solvents and DES/water mixtures. The enzymatic hydrolysis of EO was selected as a test case reaction, and five Choline Chloride (Ch-Cl) based eutectic mixtures; namely, ChCl-Ethylene glycol (EG) (1:2), ChCl-Glycerol (1:2), ChCl-Urea (1:2), ChCl-Sorbitol (1:1), ChCl-Methanol (MeOH) (1:2) were screened. Results were depicted in Fig. 6. In this experiment, under the optimized conditions, 15 mM EO and 150 µg enzyme were incubated in DESs mixtures at 30 °C for 65 min. From Fig. 5, the OD yield in these solvents system was more when reactions were incubated for 65 min than for 30 min, and ChCl-Glycerol was the optimal deep eutectic solvent for hydrolysis of EO producing 9.1 mM OD in optimized reaction conditions with a yield of 60.5%. Yleh was able to hydrolyze the EO in other deep eutectic mixtures containing ethylene glycol (OD yield 55%), urea (OD yield 49.4%), sorbitol (OD yield 48%), and methanol (OD yield 41.4%), providing a better reaction media than the conventional organic solvents.

### Absolute configuration of epoxide 1,2-epoxyoctane and diol 1,2-octanediol

The absolute configuration of extracted remaining epoxide and reaction product diol from the hydrolysis reaction were determined using Polarimeter as follows:

epoxide (*S*)-1,2-epoxyoctane: [α]^20^ D = -1.5 (c = 0.20, pentane).

diol (*R*)-1,2-octanediol: [α]^20^ D = + 5.8 (c = 0.50, methanol).

These results indicated that (*S*)-1,2-epoxyoctane remained from the racemic mixture while (*R*)-1,2-octanediol was the reaction product of this hydrolysis, indicating that the enzyme is stereospecific to (*R*)-1,2-epoxyoctane.

## Discussion

In recent times interest in biocatalysis in non-aqueous media has been triggered as it provides enantio and regioselective synthesis of compounds, exclusion of water dependent side reactions and is a greener route as compared to chemical synthesis using less hazardous compounds and ambient reaction conditions. Enzymes stable in such non-aqueous systems such as organic solvents, ionic liquids, sub and supercritical fluids and deep eutectic solvents are of industrial relevance. Studies suggests that factors such as Log P value (the ratio of a compound’s concentration partitioned in organic to aqueous phases, LogP = log_10_ ( [organic] / [aqueous]), functional groups and molecular structures of solvents alter the microenvironment of the enzyme molecule thereby affecting its tertiary and secondary structure and hence its catalytic properties. For example, enzymes such as amylase, glucosidase, trypsin have shown an increase in activity after incubation with acetonitrile for 3 h (Wang et al. 2016; Steenkamp, 2021). Use of enzymes on an industrial scale is dependent on enzyme performance, production costs and reaction products that must meet commercial and industrial standards. In this regard, the easy availability of biocatalyst and optimization of reaction product using biocatalyst are of high importance. Epoxide hydrolases are important enzymes in the biotransformation of endogenous epoxy compounds and the elimination of xenobiotics. They enable highly reactive epoxides convert into less reactive corresponding diols by hydrolysis. They are prevalent in nature and have a significant impact on the development of biologically active drugs in the pharmaceutical sector. A real alternative to asymmetric organic syntheses is the use of enzymes to produce enantiopure building blocks, which may excel merely chemical methods in terms of yield, enantiomeric purity of the final product, and environmentally-safe. Such crucial chiral building components are vicinal diols and enantiopure terminal epoxides.

Epoxide hydrolases’ efficacy as biocatalysts for the synthesis of enantiopure epoxides and diols depends on a number of variables, including their accessibility, stability, activity, enantioselectivity, and regioselectivity of the enzymatic process. So far, it appears that the filamentous fungi, yeasts and bacteria produce the most remarkable and promising epoxide hydrolases (Wu et al. 2007).

The current study’s objectives were to assess the expression of the *Y. lipolytica* gene that codes for epoxide hydrolase in recombinant *E. coli*, purification and biochemical characterization of recombinant protein, optimization of EH catalysis reaction using response surface methodology and use of deep eutectic solvents during hydrolytic reaction were set and performed. The bioconversion yield was calculated using epoxide hydrolase activity of recombinant enzyme Yleh.

The *Yleh* gene was successfully cloned and expressed in *E. coli* and purified to unity. Enzymes epoxide hydrolase activity was performed spectrophotometrically and reaction product was identified as diol using GC-MS analysis. The labelled water experiment confirmed the incorporation of oxygen moiety into the diol product from the water molecule. In biochemical characterization of recombinant Yleh, effect of pH, temperature, different additives, substrate specificity, kinetic parameters were determined using spectrophotometric assay. The results showed that the most of the EHs exhibit a broad pH-activity profile with maximum catalysis occurring at pH range 6.0–9.0 with maximum activity occurring at temperature range 28–55 °C (Arand et al. 1999; Oliveira et al. 2016; Bendigiri et al. 2017; Rink et al. 1997; Elfstrom 2005). The results of additives effects on Yleh activity, the reducing agents (DTT and 2-mercaptoethanol), coenzymes (NADH and NADPH), and metal chelator (EDTA) showed no significant effects on the EH activity of Yleh, suggesting their non-involvement during enzyme catalysis, and cysteines may not play a role in enzyme activity similar to earlier reports (Oliveira et al. 2016; Bendigiri et al. 2017; Rink et al. 1997; Xue et al. 2014). From earlier reports, EHs are co-factor independent (Bendigiri et al. 2018; Lee and Shuler 2007), and the results obtained for Yleh were in line with previous reports.

From earlier reports, Ag^2+^, Hg^2+^, and Zn^2+^ salts have been severely inhibiting the EH activity of Ylehd from *Y. lipolytica* NCIM 3589, PchEHA from *P. chrysosporium* and AmEH from *Agromyces mediolanus* ZJB120203, *Rhodosporidium toruloides* EH activity (Bendigiri et al. 2017; Xue et al. 2014; Li et al. 2009; Liu et al. 2011) similar to Yleh activity. From the results obtained for the effects of additives on Yleh activity, the microenvironment of the active site pocket could be affected by the valence state of metal ions and its interaction with active site residues. Further, the substrate specificity studies were carried out using aliphatic epoxide substrates. The recombinant Yleh hydrolysed these epoxides with different rates which makes Yleh different from previously reported EHs. Moreover, the reported.

EH from *Trichoderma reseei* (TrEH) (Oliveira et al. 2016), *Solanum tuberosum* (StEH1) (Lindberg et al. 2008), *Beauveria sulfurescens* (Pedragosa-Moreau et al. 1996), *Phanerochaete chrysosporium* (PchEHA) (Zhang et al. 2011) and *Aspergillus usamii* E001 (Hu et al. 2015) preferentially hydrolyze styrene oxide. In contrast, *Agrobacterium radiobactor* AD1 (Rink et al. 1997) showed specific activity towards EO similar to EHs from yeasts such as *Rhodotorulla glutinis*, *Rhodosporidium toruloides* (Smit 2004), and bifunctional enzyme from *Y. lipolytica* (Bendigiri et al. 2017) which showed preference towards 1,2-epoxides of chain lengths C_6_-C_8_ rather than towards styrene oxide and *p*-nitro styrene oxide. PchEHA from *P. chrysosporium* could act on epoxides with varied structural scaffolds, such as styrene oxide and 1,2-epoxybutane (Li et al. 2009). For further research, we studied the kinetic properties of properties of Yleh towards EO showed that Yleh could hydrolyze the EH with higher catalytic rate than previously reported enzymes.

The bifunctional enzyme (Ylehd) from *Y. lipolytica* showed a lower catalytic constant (*k*_cat_) and catalytic efficiency (*k*_cat_/*K*_m_) of 48.28 ± 1.98 s^− 1^ and 96.56 ± 1.55 mM^− 1^ s^− 1^, respectively, for EO (Bendigiri et al. 2017). The results suggest that the high EH activity seen in this marine yeast could have evolved its EH activity for adaptation to the diverse stress conditions from natural and anthropogenic sources as the yeast has been isolated from the coastal waters off Mumbai High, an offshore oilfield polluted with oil and industrial wastes. Our earlier studies have shown the potential of this strain to degrade different components including epoxides which can induce EH activity (Vatsal et al. 2011, 2015; Bendigiri et al. 2017).

The response surface methodology (RSM) was applied to improve the Yleh protein’s enzymatic hydrolysis conditions for future study. Optimization of the hydrolytic reaction product was carried out initially by the two-level factorial design experiments, where the significant variables were found to be substrate concentration (A), enzyme concentration (B), and a two-factor interaction term (AB) of the product of enzyme and substrate concentration. Further, the data of 13 experimental runs using the three-level factorial design experiments with two independent factors, namely, substrate concentration (A) and enzyme concentration (B) and response OD in mM were obtained. The response OD is one of the important factor that affect the enzymatic hydrolysis, therefore, validation of the hydrolysis response should be performed in order to approve the predicted response generated by RSM.

From a sustainability and technological standpoint, deep eutectic solvents (DESs) have become a viable replacement for conventional organic solvents during the preceding ten years or so. Due to the vast array of structural combinations that DESs may accommodate, an ideal DES can be established for any particular enzyme reaction system. A DES can operate as a solvent or co-solvent, an extraction reagent for an enzymatic product, and as an initial solvent for enzymatic biomass in biocatalytic processes. Since lipases are among the most significant industrial enzymes, it is not unexpected that hydrolases have received the highest interest in research in DESs so far. However, there aren’t many articles discussing the synthetic processes in DESs involving various hydrolytic enzymes such as epoxide hydrolases, haloalkane dehalogenases, lyases and dehydrogenases, phospholipases, proteases, etc. (Panić et al. 2021). In this study, the synthesized Ch-Cl based DESs were employed resulting in a high yield of the product OD. The physico-chemical properties of DES have been recently reviewed and their phase behavior, polarity, density, viscosity etc. have been reported (Omar and Sadgehi, 2022; El Achkar et al. 2021). A low number of studies on effects of temperature are available. Marchel et al. (2022) reporting ChCl:urea DES revealed a high long-term thermal stability (60 to 120 °C for 2 h) with less toxicity to bacteria. Another study on choline chloride based DES using isothermal and dynamic thermogravimetric analysis/Fourier transform infrared-attenuated total reflectance spectroscopy (TGA/FTIR-ATR) techniques showed the effect of temperature on stability and structural changes of DES. The study suggests that the DES maintained their stability and structure under operational temperatures (30 to 60 °C) which were significantly lower than the onset decomposition temperatures (110 to 238 °C ) of DES (Delgado-Mellado et al. 2018). Thus, based on their report, the conditions of heating at 60–80 °C for 1 h used in our study, may not seem to affect the structural changes or stability of the DES. Additionally, El Achkar et al., (2021) reviewed that the DES’s density showed a temperature-dependent behavior; it decreased linearly with the increasing temperature. Another report (Smith et al. 2014), demonstrated that the bond between the choline cation and its corresponding HBD became weaker as the temperature raised. The mobility of the entire system can be significantly altered by the HBD’s molecular structure and the choline cation diffuses more slowly than the corresponding HBD in ChCl:EG, ChCl:glycerol, and ChCl:urea.

In addition, Deep Eutectic solvents were used to optimize the production and yield of the product OD. DES containing systems are a group of biodegradable ionic liquids, less toxic and easily prepared in pure states from cost-effective starting materials and sustainable solvents. DES can be used in enzyme reactions having a hydrophobic substrate with limited aqueous solubility. DES can be made by mixing the hydrogen bond acceptor (HBA), such as choline chloride (ChCl), with a hydrogen bond donor (HBD), like amide, amine, alcohol, or carboxylic acid (Paiva et al. 2014; Abbott et al. 2003; 2004) and used as a greener alternative to organic solvents. DES has been effectively used for reactions carried out by soybean epoxide hydrolase (Cao et al. 2016), potato epoxide hydrolase (Lindberg et al. 2010), Phenolic acid decarboxylase (Schweiger et al. 2019), lipase (Durand et al. 2012), protease (Cao et al. 2015), etc. Cao et al. (2022) studied the hydrolysis efficiency of *Candida antarctica* lipase B (CALB) and β-Glucosidase (β-GC) in the presence of different DESs, and results showed that the efficiency of the hydrolytic reaction was altered by the substrate solubility and its diffusion in DESs. DESs elements could increase the hydrogen bonding interactions with surface amino acid residues of enzymes to build the enzyme-DES complex. The role of DESs elements in altering the native conformation and activity of enzymes is under investigation. Lindberg et al. (2010) have previously used choline chloride-based deep eutectic mixtures as co-solvents for epoxide hydrolase catalyzed hydrolysis of a racemic (1,2)-trans-2-methylstyrene oxide. Cao et al. (Cao et al. 2016) have used the immobilized soybean epoxide hydrolase as an efficient biocatalyst (SEH@UiO-66-NH2) for asymmetric hydrolysis of EO to (*R*)-OD in a deep eutectic solvents-containing system with improved yield (41.4%). Dolcet et al. (Dolcet et al. 2018) achieved a yield of 21.4% of (*R*)-1,2-octanediol from *rac*-1,2-epoxyoctane (9 mM) using *Aspergillus terreus* strain’s lyophilized mycelium (10 mg) in 6 h reaction time.

In addition, we also determined the absolute configuration of the reaction product OD and retained substrate EO in the hydrolytic reaction using Polarimeter. As shown in the previous work by Weijers et al. (1998), using aliphatic epoxides as substrates and *Rhodotorula glutinis* cells as a source of enzyme the absolute configurations of product diols and residual substrates were determined. These reactions also produced (*R*)-diols as a product and residual (*S*)-enantiomers of epoxides.

In conclusion, chiral 1,2-epoxyoctane and its vicinal diol 1,2-octanediol are central building blocks to produce many pharmaceutically and industrially important bioactive compounds. Enzymatic catalysis provides an eco-friendly and sustainable approach to producing compounds like OD. In this study, the reaction conditions for the biocatalytic hydrolysis of EO by Yleh, an epoxide hydrolase, for the production of OD were optimized using a statistical design of experiments at the lab scale. Additionally, the solubility of EO was addressed using a mixture of eco-friendly deep eutectic solvents (choline chloride and glycerol) under the optimized conditions, obtaining a high yield of 9.1 mM. Thus, the effective hydrolysis of EO by Yleh was demonstrated and can suitably modify the reaction for upscaling and larger-scale productions.

## Electronic supplementary material

Below is the link to the electronic supplementary material.

Supplementary Material 1

## Data Availability

The data sets used for the current study are available from the corresponding author upon reasonable request.
