# Supplementary material for: Potential of Y. lipolytica epoxide hydrolase for efficient production of enantiopure (R)-1,2-octanediol
Source: AMB Express. 2023 Jul 26;13:77. doi: 10.1186/s13568-023-01584-1 (PMC10371975; doi:10.1186/s13568-023-01584-1)
Supplement: Supplementary file 1 — Supplementary Material 1 [file 13568_2023_1584_MOESM1_ESM.docx]

**Supplementary Information**

**Title**: Potential of *Y. lipolytica* epoxide hydrolase for efficient production of enantiopure (*R*)-1,2-octanediol.

**Authors:** Vijaya P. Godase^a,b^, V. Ravi Kumar^c^, Ameeta Ravi Kumar^a^*

**Supplementary Experimental**

*Cloning, expression and purification of Yleh*

The cloning, overexpression and purification of Yleh were carried out. In brief, the 1065 bp ORF corresponding to Yleh protein was cloned into a pET28a(+) plasmid (Novagen, USA) using the insert specific primers (Forward:5'ATGGCTAGCATGACCGACTCCCTCTCGCTCG3' and

Reverse: 5'CACCTCGAGTTACAACTTCGACTGGGAACC3') with *Nhe* I and *Xho* I restriction enzymes restriction sites. PCR program was as follows: 94 °C for 5 min, followed by 30 cycles of 94 °C for 45 s, 62 °C for 45 s, and 72 °C for 1 min with a final step at 72 °C for 10 min. The PCR amplicon was ligated in the vector and used to transform competent *E. coli* BL21AI cells. The cells were grown in Luria Bertani media supplemented with Kanamycin (50 μg mL^-1^) and induction was carried out with 1.0 mM IPTG at 30 °C. After ultrasonication, the crude extract was obtained by resuspending the cell pellet in Buffer A (50 mM Tris-sulfate buffer, pH 8.0, 100 mM sodium sulfate, 5% glycerol). The soluble fraction was loaded on the Ni-NTA affinity column equilibrated with Buffer A. The column was washed with Buffer B (50 mM Tris-sulfate buffer, pH 8.0, 100 mM sodium sulfate, 5% glycerol, 10 mM Imidazole). The Yleh protein bound to the column was eluted with elution Buffer C (50 mM Tris-sulfate buffer pH 8.0, 100 mM sodium sulfate, 5% glycerol, 200 mM Imidazole). The eluted protein dialyzed against Buffer D (50 mM Tris-sulfate buffer pH 8.0, 100 mM sodium sulfate, 5% glycerol, 1 mM DTT) followed by gel filtration on a Superdex S200 10/300GL (Wipro GE Healthcare, India) equilibrated with Buffer E (50 mM Tris-sulfate buffer pH 8.0, 100 mM sodium sulfate, 2.5% glycerol). The purity of the Yleh protein was evaluated by SDS-PAGE (12%) under reducing conditions and by Native PAGE (10%) under non-reducing conditions.

*Effect of pH, temperature, additives on Yleh activity*

The effect of pH (pH range 2.0 to 11.0) on enzyme activity of recombinant protein Yleh was determined using following buffers (50 mM each): glycine-HCl buffer (pH 2.0), sodium acetate-acetic acid buffer (pH 4.5), sodium phosphate buffer (pH 6.0), Tris-sulfate buffer (pH 8.0) and glycine-NaOH buffer (pH 10.0 and 11.0). The enzyme stability at different pH was determined by incubating the enzyme solution with 50 mM buffers at pH 2.0-11.0 at 4 °C for 60 min. For temperature optima, EH activities were measured at temperature ranging from 20-50 °C and assayed at pH 8.0. The enzyme’s thermostability was determined by pre-incubating the enzyme in water bath at above mentioned temperatures for 60 min. The maximal enzyme activity was considered 100%, and the relative enzyme activity (%) was estimated.

The effect of metal salts, metal chelators, reducing agents, and coenzymes (1 mM) on the epoxide hydrolase activity of Yleh was also determined using 1,2-EO as substrate. In brief, 2 µg purified Yleh was incubated with different additives in 50 mM Tris-sulfate buffer, pH 8.0, for 30 min at 4 °C, and determined enzyme activities. The untreated enzyme activity was considered control (100%), and the relative enzyme activity (%) was calculated. All the assays were performed in triplicates, unless otherwise mentioned.

**Supplementary Results**

*Cloning, expression and purification*

The *Yleh* gene (1065 bp ORF) coded for a 354 amino acid polypeptide from *Yarrowia lipolytica* NCIM 3589 was successfully cloned and expressed in *E. coli* BL21AI in a soluble form. The 6XHis-tagged fusion protein was purified by affinity column chromatography followed by size exclusion chromatography (SEC). The recombinant Yleh protein eluted as a single peak on the S200 column at 14.19 ml and a single band was seen on both native and SDS-PAGE with molecular mass of ~84 and ~42 kDa , respectively (Fig. S1). Further, as shown in Fig S1b, the calculated apparent molecular weight of the protein by SEC was 84.1 kDa (K_av_ = 0.35) suggesting that Yleh existed as a dimer.

**Supplementary Figures**


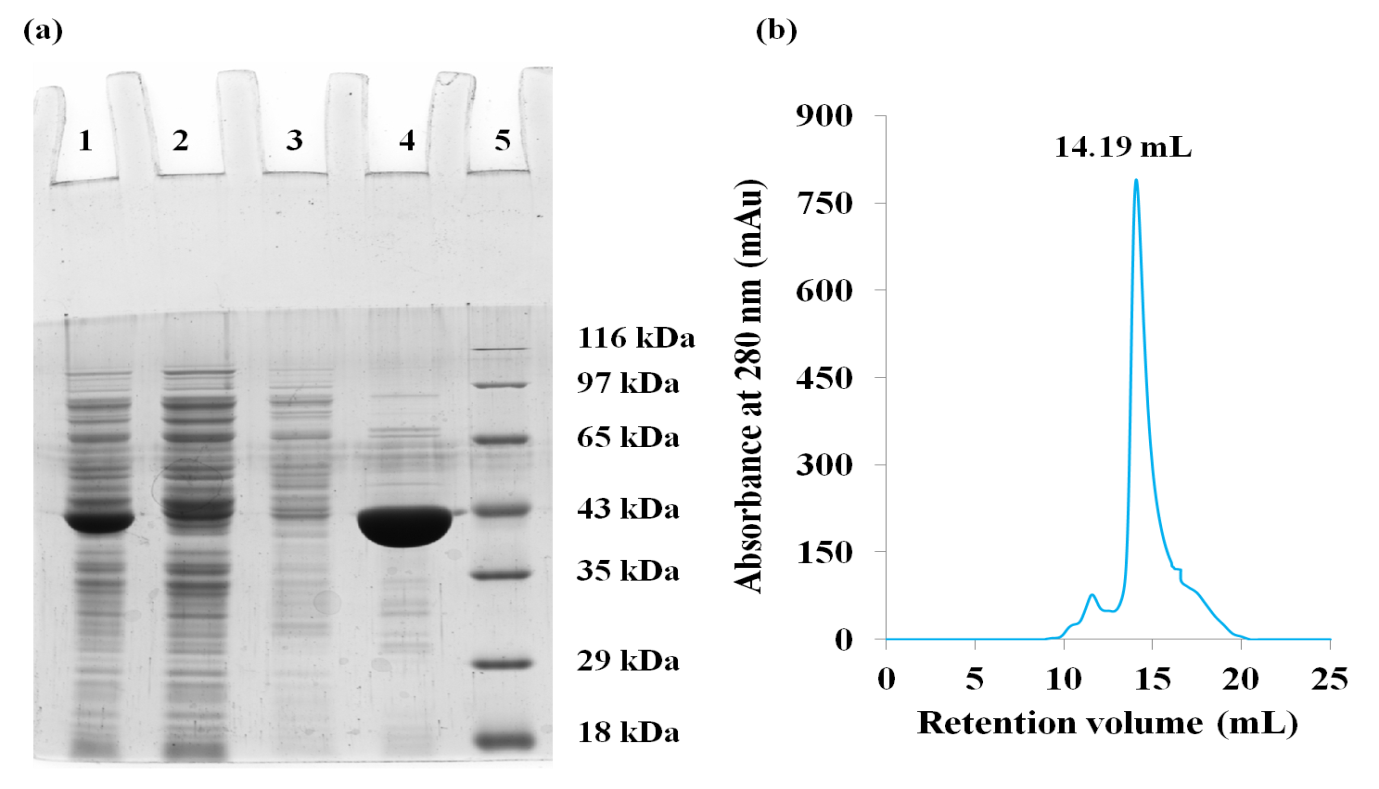


**Fig. S1.** Yleh expression and purification. (a) SDS-PAGE: Lane 1, Crude extract; Lane2, Flow through; Lane 3, Wash; Lane 4, Ni-NTA elute; Lane 5, Protein Molecular Weight Marker. (b) Size Exclusion Chromatogram of purified Yleh protein. Recombinant Yleh protein eluted as a single peak on Superdex S200 Increase column at 14.19 mL corresponding to a molecular weight of 84.1 kDa.


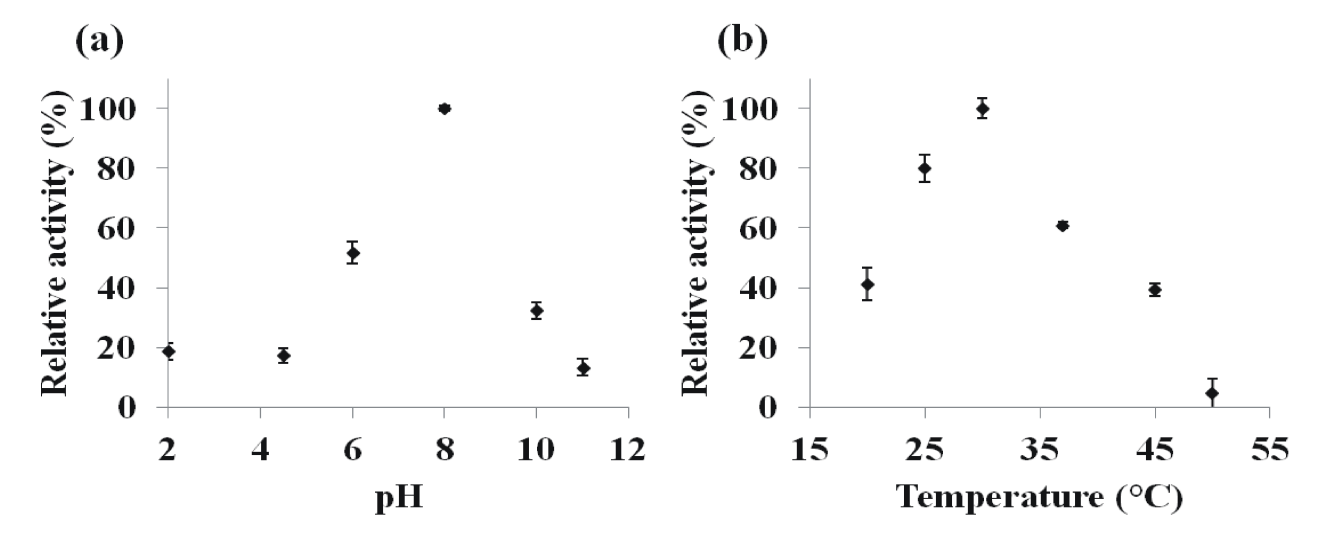


**Fig. S2.** Effect of pH and temperature on the activity of Yleh. (a) pH profile showing optimum at 8.0. All assays were performed at 30 °C using different buffers at different pH values (solutions in a range of pH from 2.0 to 11.0) with 1, 2-Epoxyoctane as the substrate. (b) Temperature profile showing an optimum temperature at 30 °C. All assays were performed with Tris-sulfate buffer, pH 8.0 at different temperatures (ranging from 20 to 50 °C) with 1,2-Epoxyoctane as the substrate. All the assays were performed thrice in triplicates and results expressed as means (±SEM).


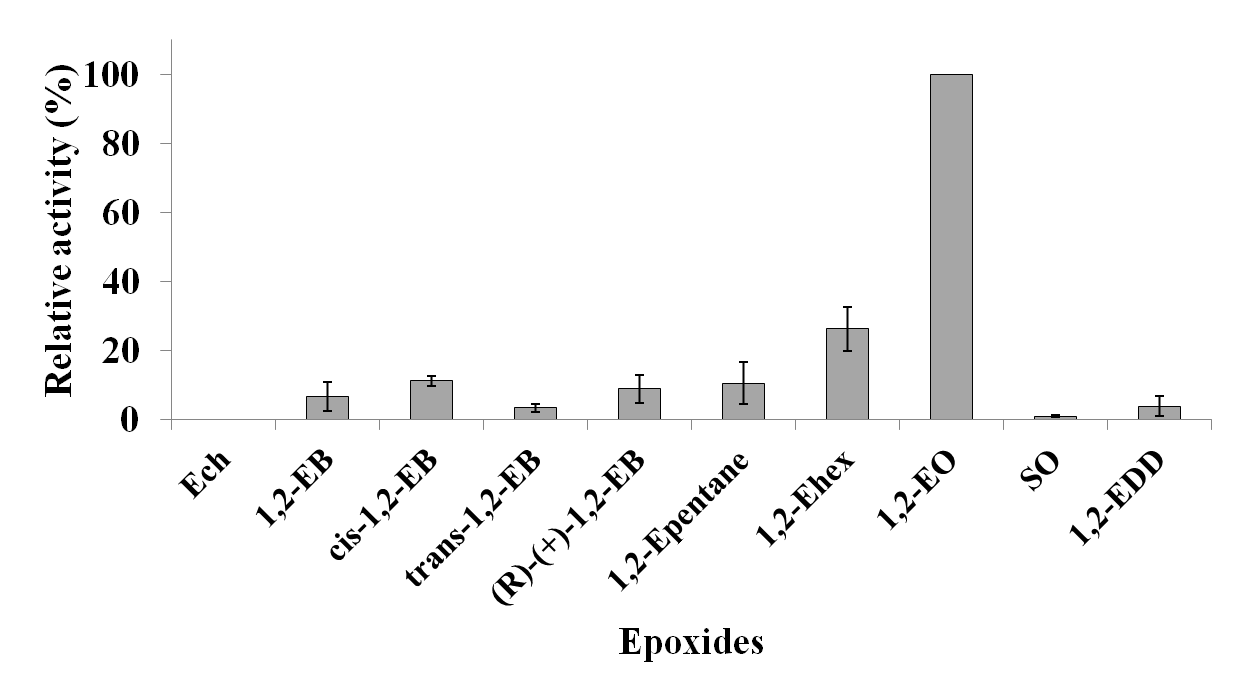


**Fig. S3** Substrate specificity of Yleh. All values expressed as relative activity (%) with respect to EO. All the assays were performed thrice in triplicates and results expressed as

means(±SEM)

**Fig. S4.** Determination of kinetic parameters of Yleh with 1,2-EO as substrate.

(A) MM plot. (B) LB plot. (C) Hill Plot. All the assays were performed thrice in triplicates and results expressed as means (±SEM)

**Table S1 Effect of additives on hydrolytic reaction of Yleh enzyme with 1,2-EO.**

| **Additives** | **Relative Activity (%)^a^** |
| --- | --- |
| Cu^2+^ | 150 |
| Cu^3+^ | 120 |
| Fe^3+^ | 100 |
| Fe^2+^ | 74 |
| Mg^2+^ | 108 |
| Ca^2+^ | 80 |
| Ni^2+^ | 80 |
| Ag^2+^ | ND |
| Mn^2+^ | 100 |
| Zn^2+^ | ND |
| Hg^2+^ | ND |
| EDTA | 96 |
| 2-ME | 91 |
| DTT | 96 |
| NAD | 120 |
| NADH | 100 |
| NADP | 93 |
| NADPH | 100 |
| Untreated (control) | 100 |

ND: Enzyme activity not detected under the given assay conditions.

^a^ The enzyme activity was determined under the given assay conditions using 1,2-epoxyoctane as substrate after incubating the Yleh enzyme with different reducing agents, metal chelators, metal salts and coenzymes 30 °C for 30 min. The enzyme activity in the absence of metal ions (control) was stated as 100%. All the assays were performed thrice in triplicates and average results were mentioned here. The data found Significant at 1% and 5% level of significance (p < 0.01 and p < 0.05).
